# Extreme Silica Optical Fibre Gratings

**DOI:** 10.3390/s8106448

**Published:** 2008-10-20

**Authors:** John Canning, Michael Stevenson, Somnath Bandyopadhyay, Kevin Cook

**Affiliations:** 1 Interdisciplinary Photonics Laboratories/ School of Chemistry, University of Sydney, Australia; E-Mails: j.canning@usyd.edu.au; m.stevenson@usyd.edu.au; k.cook@usyd.edu.au; 2 Fibre Optics Laboratories / Central Glass and Ceramics Research Institute, Kolkata, India; E-Mail: somnath@cgcri.res.in

**Keywords:** Regenerated gratings, fibre Bragg gratings, temperature sensing

## Abstract

A regenerated optical fibre Bragg grating that survives temperature cycling up to 1,295°C is demonstrated. A model based on seeded crystallisation or amorphisation is proposed.

## 1. Introduction

Recently, we have reported for the first time regenerated fibre Bragg gratings in standard photosensitive boron codoped germanosilicate optical fibre that approach 10% transmission [1, 2]. Regenerated Bragg gratings are gratings that have grown through thermal processing at high temperatures (∼900°C) after the seed grating written by UV light is erased. In that work we showed that these gratings could be temperature cycled with very little degradation from room temperature to ∼1,000°C. Such ultra high temperature gratings are extremely important for high temperature monitoring applications including high power fibre laser operation and numerous applications in the mining and energy industries. In particular, high temperature industrial processing of materials such steel and aluminium in smelters, can benefit from greater power consumption efficiencies if temperatures above 1,000°C are able to be monitored. In this report, using a modified ultra high temperature micro heater we demonstrate that these types of gratings can operate as high as 1,295°C with no evidence of any decay after cycling. This record temperature means these gratings can even outperform the optical fibre itself, which has become brittle. Even when the fibre breaks into pieces the grating remains intact. Such performance cannot be explained by simple chemical diffusion alone and no other silica fibre grating technology can perform in this way. An alternative model based on crystallisation (or amorphisation) of the vitreous state is proposed.

## 2. Results

**Figure 1.**
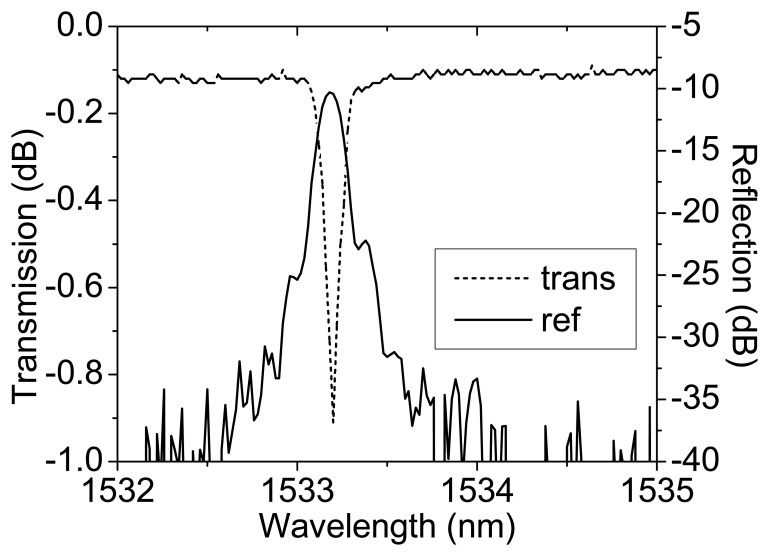
Transmission and reflection spectra for the seed grating used in this work.

Gratings (L = 5 mm) were inscribed by standard direct grating writing using an ArF exciplex laser (193 nm, τ_w_ = 15 ns, *f_pulse_* = 40-70 mJ/cm^2^, rep. rate = 10 Hz, *f*_cumulative_ = 360 J/cm^2^). The fiber used is a B/Ge-doped highly silica fiber developed at the University of Sydney (core: ∼20 mol% B_2_O_3_; ∼33 mol% GeO_2_; inner cladding: ∼11 mol% P, <4 mol% F). Boron is added to lower the refractive index of high concentration germanate doped cores to match standard telecommunications fiber whilst making the fibre highly photosensitive. It was loaded with hydrogen (180 atm, 24 hrs). The fibre is placed under tension with a standard load ∼100 g. The transmission and reflection spectra of the ordinary type I gratings (those where the refractive index is largely dictated by polarisability changes from exciation into glass defects [3]) used in this work is shown in Figure 1. As noted in [1, 2] no regenerated gratings were observed without hydrogen loading which suggests that hydrogen plays a critical role, most likely through the formation of OH.

**Figure 2.**
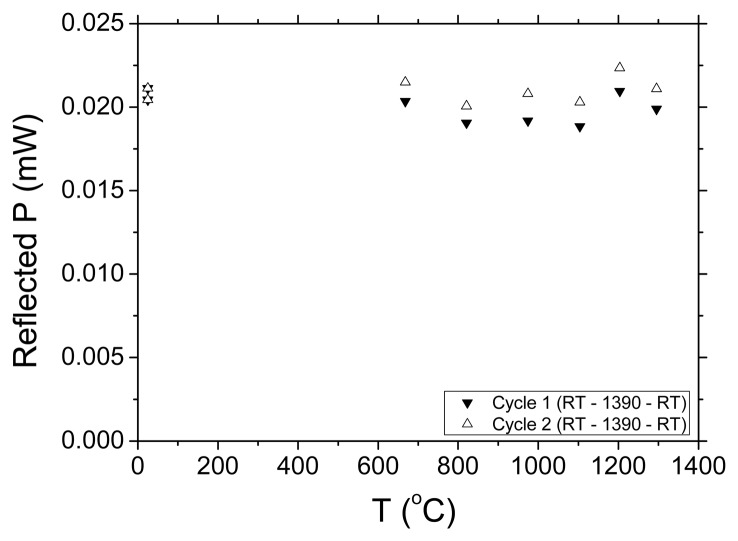
Measured reflected power during high temperature cycling two times (room temperature to 1295°C).

**Figure 3.**
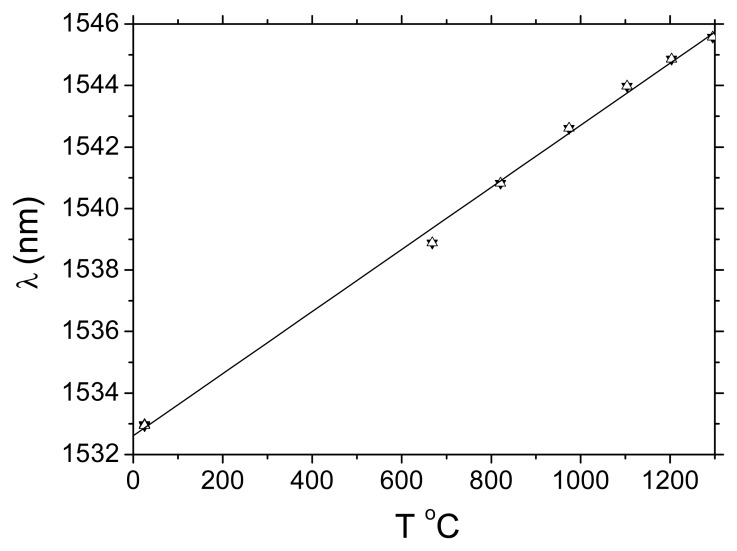
Bragg wavelength shift as a function of temperature cycling (room temperature to 1295°C). Two cycles are shown with no evidence of distortion arising from material changes.

We followed the same preparation procedure for regeneration described in [1] but without any pretension on the fibre (load <10 g). A high temperature microheater with a 1 cm hot zone was used to anneal the 5 mm long Type I grating (20 dB, L = 5 mm, written ArF 193 nm laser, H_2_ loading ∼190 atm) and regenerate a new grating ∼900°C of ∼ 10% rejection in transmission. Once a regenerated grating of a few per cent transmission was formed, it was pre-annealed to stabilise index change at 1,000°C. Since this fibre is known to become quite brittle after heating to 1,200°C, we were interested in determining whether the grating annealed out close to this temperature. The microheater current configuration was modified to allow it to exceed its previous upper limit of 1,000°C and reach a temperature as high as 1,295°C. The grating was in fact stepped through temperatures from room temperature (RT = 25°C) to 1,295°C and back to RT. This cycle was repeated twice. A summary of the results are shown in Figures 2 and 3. Two parameters were measured: the total reflected power and the Bragg wavelength shift.

Figure 2 shows the reflected power versus temperature - the total power is, within experimental error, constant indicating no degradation of the grating strength at all. The observed variations can be explained as arising from differences in thermal expansion coefficient between core and cladding and the resultant stresses. In our previous work there was an indication from the pre-tension that the grating may not be collocated in the core with the initial seed grating [1, 2]. Rather, it may be at the interface with the inner cladding, which is composed of deposited silica layers with a small quantity of both P and F – the relationship needs further investigation. Upon return to room temperature there is a full recovery. The Bragg wavelengths shift observed in Figure 2 is consistent with the previous results and, remarkably, despite approaching the softening point of the glass, there are no surprises all the way to 1295°C. A linear fit is sufficient and there is no obvious nonlinear behaviour observed outside of experimental error suggesting no obvious non-linear glass softening process or phase change.

After this point, the fibre had become extremely brittle and upon removal shattered into several pieces. The broken end still attached to the input fibre retained most of the grating – it was re-examined in reflection and found to be unchanged. Visual examination ∼24 hrs later was unable to detect any grating structure that may arise from fracturing and no obvious damage was visible. Therefore, we conclude the grating spectra remained the same despite the brittleness and this break. This suggests the induced periodic changes from regeneration are extremely stable, probably more so than the glass itself. Despite the brittleness no obvious softening point is observed in the wavelength shift. It therefore seems highly unlikely that a simple chemical diffusion model alone can explain the cycling behavior at such high temperatures.

## 3. Discussion

In light of the experiments, the brittleness together with the possibility the grating is in the cladding and therefore not dependent directly on any core dopants, a model of crystallization is proposed. It is known that vitreous silica can crystallize at high temperatures and high pressures. The high temperature is the required 900°C whilst the pressure is that which arises from the differences in thermal expansion coefficient between the core and cladding at such temperatures, which is enhanced by the induced periodic UV stress profile of the Type I grating – the irradiation of germanosilicate optical fibres with 193 nm has been shown to increase the core-cladding interface stress by as much as 100 MPa [4] to lead to pressures above 250 MPa, providing some insight to the pressure regimes capable of being reached within optical fibres with and without irradiation. Under such conditions, the most likely phase of silica that is sufficiently close in structure and in index to vitreous silica and only slightly higher in terms of the softening point is the crystalline polymorph cristabolite, the properties of which are compared to vitreous silica in Table 1. It is slightly higher in m.p. but notably its density is higher by ∼0.07. To explain the grating, the initial Type I grating is formed from a UV-induced periodic modulation of OH and local structure that gives rise to periodic variation in the stress at the interface between core and cladding. OH formation in glass is known to exert a dilatory hydrostatic internal pressure within glass so in the regions where OH is formed we can expect stress relief in typical fibres where there is tensile stress on the core from the cladding (noting that this depends on how an optical fibre is drawn during manufacture). Consequently, heating to very high temperatures leads to a periodic variation in pressure and therefore stress that is sufficient to seed crystallization in a periodic fashion probably at the interface. Were it across the core, the relatively large index change expected should lead to much stronger gratings. Therefore, either the overlap of the optical mode is small since the change is largely at the interface, or the effective fringe contrast is poor which is also feasible. Further experiments involving annealing of different strength gratings show a correlation between regenerated grating strength and seed grating strength. This is the subject of further work.

## 4. Conclusions

We have demonstrated fibre Bragg grating performance up to 1295°C. The grating survived temperature cycling even thought the fibre itself changed properties and became brittle. We propose that this can be explained on the basis of a change of phase of the glass in the periodic regions which is more stable than the vitreous state. An example is crystallisation of cristabolite arising in part due to very high local pressures formed during hydrogen reaction in the glass along with very large stresses.

## Figures and Tables

**Table 1.** Properties of vitreous silica and cristabolite.

	**Vitreous silica**	**Cristabolite**
**m.p. (°C)**	1713	1722
**Density (g.cm^-3^)**	2.196	2.265
